# Extracting user influence from ratings and trust for rating prediction in recommendations

**DOI:** 10.1038/s41598-020-70350-1

**Published:** 2020-08-12

**Authors:** Wenchuan Shi, Liejun Wang, Jiwei Qin

**Affiliations:** Key Laboratory of Signal Detection and Processing, Xinjiang Uygur Autonomous Region, Xinjiang University, Urumqi, 830046 China

**Keywords:** Computational science, Computer science, Information technology

## Abstract

The Collaborative Filtering (CF) algorithm based on trust has been the main method used to solve the cold start problem in Recommendation Systems (RSs) for the past few years. Nevertheless, the current trust-based CF algorithm ignores the implicit influence contained in the ratings and trust data. In this paper, we propose a new rating prediction model named the Rating-Trust-based Recommendation Model (RTRM) to explore the influence of internal factors among the users. The proposed user internal factors include the user reliability and popularity. The internal factors derived from the explicit behavior data (ratings and trust), which can help us understand the user better and model the user more accurately. In addition, we incorporate the proposed internal factors into the Singular Value Decomposition Plus Plus (SVD + +) model to perform the rating prediction task. Experimental studies on two common datasets show that utilizing ratings and trust data simultaneously to mine the factors that influence the relationships among different users can improve the accuracy of rating prediction and effectively relieve the cold start problem.

## Introduction

The past decades have witnessed a burgeoning Internet throughout the world. And the social rating website has become a major platform for people to share their personal experience and satisfaction with products or services with others. This information presents new opportunities for improved Recommendation Systems (RSs) and further expands the research scope of researchers to explore user preferences.

Among numerous recommendation methods, the Collaborative Filtering (CF) algorithm^1–3^, which only takes advantage of ratings to predict user preferences and achieve a recommendation list for a specific user, has attracted extensive attention from researchers. Due to the characteristics of the data sparsity and uneven data distribution in the rating, an increasing number of researchers have begun to introduce user trust into recommendation algorithms to solve the problems of low recommendation accuracy and cold start. The social information^4–9^ and user interaction^10,11^ provide relevant information between different users, and many methods centering on the user trust have shown that they perform well in RSs, which can solve the cold start problem effectively. Whether there is a social relationship between users often depends on whether the users trust each other in social networks, and the social relationship provides user preference information to some extent. In addition, the trust value between users is usually in a binary form^12–14^, which is the same as the format of the user-item ratings matrix. If there is a trust relationship between two users, the padding value of the corresponding position of the user-user matrix is "1", otherwise it is "0". According to the theory of social relations, users with strong social relations tend to have similar preferences and influence in some aspects. However, the existing methods^15–17^ have mainly studied the trust between different users based on the objective auxiliary information of users. Table 1. represents the ratings on the movies. There are many movie-rating sites where users can express their satisfaction degree (in the form of the ratings) on the movies they have seen before. As shown in Table 1., the left side is the movie name and year, and the right side is the overall rating of all users who have watched the movie. Because the above usable information lacks the relationship between different users, the cold start problem exists. Therefore, mining the internal factors based on user ratings and trust has become a topic of interest in RSs. For example, if a user sees a high rating for a movie posted by someone he or she trusts, he or she will better decide to watch the movie or not.

**Table 1:** An example of user ratings on the movies.

Movie Name (Year)	User Star
The Shawshank Redemption (1994)	9.2
The Goldfather (1972)	9.1
The Goldfather: Part II (1974)	9.0
The Dark Knight (2008)	9.0
12 Angry Men (1957)	8.9

Thus, a new Rating-Trust-based Recommendation Model (RTRM) is proposed in this paper. To improve the accuracy of the rating prediction and alleviate the cold start problem, the proposed model discusses the two internal factors that affect the relationship between different users, namely, user reliability and popularity. Compared with previous work^18–21^, the main contributions of this paper are as follows:

- We propose the RTRM that incorporates the users’ internal factors into Matrix Factorization (MF) to improve the accuracy of rating prediction and alleviate the cold start problem.
- We propose using user reliability and popularity as user internal factors on social impact. User reliability extracted from the user rating represents that the larger the span and the number of his or her ratings, the higher his or her reliability, and the greater their influence on the others. In addition, the user popularity extracted from the user trust indicates that the more others trust the user, the higher their popularity and the greater their influence on others.
- We conduct experimental studies on two datasets, and the experimental results supported the effectiveness of our model in improving the accuracy of rating prediction and alleviating the cold start problem.
- We discuss the four factors that may affect the performance of the RTRM model. The factors include: the Potential Vector Dimension K of Users and Items, the Factors Between Users, the Density of Factor Matrix ${{\varvec{C}}}_{{\varvec{u}},{\varvec{v}}}$ and the Training Data Volume.

The rest of this paper is organized as follows: we first summarize the work related to RSs and social recommendations in Sect. 2. Then, Sect. 3 and Sect. 4 detail our proposed RTRM. Finally, we present the experimental results and analysis in Sect. 5, Sect. 6 and summarize the paper in Sect. 7.

## Related work

###  Recommendation systems

Among the many recommendation methods, the CF algorithm has been widely studied in industry and academic circles and has achieved great success. The CF algorithm, which is based on MF^22^, presents user ratings for an item in the form of a matrix, mines the low-dimensional hidden feature space, and represents the users and the items in the low-dimensional space. Then, it describes the correlation for the users and the items by the inner product between the user vectors and the item vectors. However, the rating matrix has the characteristics of highly sparse data and uneven distribution, which further lead to the problems of low recommendation performance and cold start^23,24^. Cai et al.^25^ studied the CF algorithm from a different perspective and proposed a new CF algorithm based on typicality. This algorithm can achieve good rating prediction performance, even in a sparse data environment.

To further improve the accuracy of the rating prediction in recommendation algorithms, researchers have developed many new models on the basis of MF and achieved good effects to a certain extent, such as Non-negative Matrix Factorization^26^, Fast Maximum Margin Matrix Factorization^27^, Probability Matrix Factorization (PMF)^28^, Singular Value Decomposition (SVD)^29^, and Localized Matrix Factorization^30^. The above models aim to learn the potential factors of the users and items from the rating matrix to make rating predictions and generate personalized recommendation lists. However, the MF, which only relies on rating data, suffers from the cold start problem when new users make recommendations.

### Social recommendation systems

Researchers have addressed the cold start problem on the above recommendation models by introducing additional information, among which social information was the most commonly used. The MF-based recommendation model has become an important basic method for constructing the social recommendations because of its good expansibility and flexibility. The social information provides the users with relevant information, and the article content description and review information add useful information guarantees^31^ for the connection between the items and have obtained good recommendation results to some extent in models such as SocialMF^32^, ContextMF^33^, TrustMF^34^, PRM^35^ and TrustSVD^36^. In addition, some recommendation models based on trust calculate the similarity between users to represent the trust value and thus improve the accuracy of the recommendation. Sato et al.^37^ used knowledge related to probability and statistics to predict the personal preferences of users and their influence on other users. Yang et al.^38^ proposed the concept of a "trust circle" based on the PMF in a social network. However, the user categories and tag information used in social networks are not always directly available.

The above recommendation models based on MF all use trust relations to realize recommendations. In recent years, some scholars have proposed using the social network analysis method to find indirect social relations among users to construct better recommendation algorithms. Zhang et al.^39^ proposed to extract the implicit and reliable social information from users’ feedback and identify the top-k semantic friends for each user to alleviate the issue of data sparsity and cold start in RSs. Jiang et al.^40^ introduced the network embedding technology into rating prediction task which based on user trust information, combined with social trust data and rating data, and represented the items with low-dimensional vector, proposed a new trust embedding framework. Li et al.^41^ proposed a recommendation model (MFC) for the regularization and fusion of social information in overlapping communities. Jiang et al.^42^ proposed a slope one algorithm based on the fusion of trusted data and user similarity, which improved the prediction accuracy of the original algorithm. The CESNA model proposed by Yang et al.^43^ obtains users' community membership information from social networks and classify them into users in the same community with similar tastes. Wang et al.^44^ calculated the quantitative trust value between users, combined the user trust with the CF algorithm, and proposed a recommendation method based on trust transfer to achieve effective recommendation. Tang et al.^45^ proposed a recommendation model named SoDimRec on the basis of the social dimension, which was based on the simple assumption that social relations have isomorphisms based on the existing recommendation models. Jin et al.^46^ proposed a hybrid optimization CF algorithm based on the user characteristics and trust, which can improve the recommendation accuracy of the RSs and effectively alleviate the problems of cold start and data sparsity. The existing recommendation methods that use indirect social relations usually adopt a segmented strategy: first, the user groups are mined based on pure trust, and then user recommendation implicit variables are modified with group information. However, user groups only rely on user trust when they are found and ignore the influence of user preferences contained in user ratings on user group identification.

In recent years, many researchers have begun to extract user preferences based on user emotions. User comment information is the most common research object. Generally, user comment information is usually divided into two categories: positive and negative. Chen et al.^47^ believed that the users' attention will affect their consumption decisions and thus affect the performance of the RSs. So they came up with a probabilistic model that takes into account both user concerns and preferences. Ganu et al.^48^ used free-form text comments to identify topics and user emotion information and clustering technology to gather similar users together to improve user experience. Lei et al.^49^ tried to use emotion analysis to extract the characteristics of users and items to carry out personalized recommendations for users. Chen et al.^50^ studied how to use comment content to improve the user experience based on the content, the CF and the preference recommendation. Lou et al.^51^ attempted to classify the user's entire comment sentiment as positive, negative, or neutral sentiment polarity. Zhao et al.^52^ studied the emotional bias and evaluation reliability of users to measure their impact on specific fields. The above models, which are based on user comment information to analyze user emotion, have achieved measurable results in recommendation performance. However, there are problems such as a large amount of data and the low reliability of user comment information.

### Problem formulation

The symbols used in this paper and their meanings are given in Table 2. The proposed Rating-Trust-based Recommendation Model (RTRM) employs the SVD +  + framework to combine user reliability and user popularity as two products from user-item rating and user-user trust respectively, taking the best of each to improve the recommending performance. In particular, the user reliability ${{D}}_{{u}}$ takes the user-item rating as input, and describes the fact that users who commonly rated higher on a certain item shared the same interest to some extent based on the historical user-item interactions. Similarly, the user popularity ${{H}}_{{u}}$ indicates the knowledge that the users who trust a particular user have the same tastes and opinions to some extent based on user-user trust in the social network. It's worth noting that: the trust and popularity are non-orthogonal, and the ratings and reliability are the same. There is a group of users ${\text U} = \left\{{{u}}_{1},{{u}}_{2}{,\cdot \cdot \cdot ,}{{u}}_{{M}}\right\}$ and a set of items ${\text I} = \left\{{i}_{1},{i}_{2},\cdot \cdot \cdot ,{i}_{N}\right\}$ in the dataset. The rating of the user for the item is expressed by the rating matrix ${{R}}_{{M}} \times {{N}}$, where ${{R}}_{{u,i}}$ represents the rating of user *u* for item *i*, which is usually an integer in a range between 1 and 5. The social relationship information ${{O}}_{{M}} \times {{M}}$, where ${{O}}_{{{u,} \, {{u}}}^{{\prime}}}$ represents the trust value of user *u* to another user ${{u}}^{{\prime}}$, which is usually 0 or 1. In the social relationships information, we extract the user reliability ${{D}}_{{u}}$ and user popularity ${{H}}_{{u}}$ among users from user ratings and trust. The ${{C}}_{{u,v}}$ is determined by ${{D}}_{{u}}$ and ${{H}}_{{u}}$, indicating the influence of user *u* on user *v*. The main task of our proposed model is to predict user *u*'s rating of unknown item *i*, ${{P}}_{{M}} \times {{K}}$ represents the potential user feature matrix, and ${{Q}}_{{K}} \times {{N}}$ represents the potential item feature matrix.

**Table 2:** Symbols and their description.

Symbol	Description	Symbol	Description	Symbol	Description	Symbol	Description
${{U}}$	A set of users	${{I}}$	A set of items	${{C}}_{{u, v}}$	User u’s influence on user *v*	${{O}}_{{M}} \times {{M}}$	The social trust matrix
${{M}}$	User numbers	${{N}}$	Item numbers	${{D}}_{{u}}$	User u’s reliability	${{H}}_{{u}}$	User u’s popularity
${{R}}_{{M}} \times {{N}}$	The rating matrix	${\widehat{{R}}}_{{M}} \times {{N}}$	The predicted rating matrix	${{J}}$	The objective function	$\lambda ,\mu ,\gamma$	The parameters in the objective function
${{P}}_{{M}} \times {{K}}$	The user latent feature matrix	${{Q}}_{{K}} \times {{N}}$	The item latent feature matrix	${{UMI}}$	The user-wise mutual information	${{K}}$	The user and item potential feature dimensions

**Figure 1:**
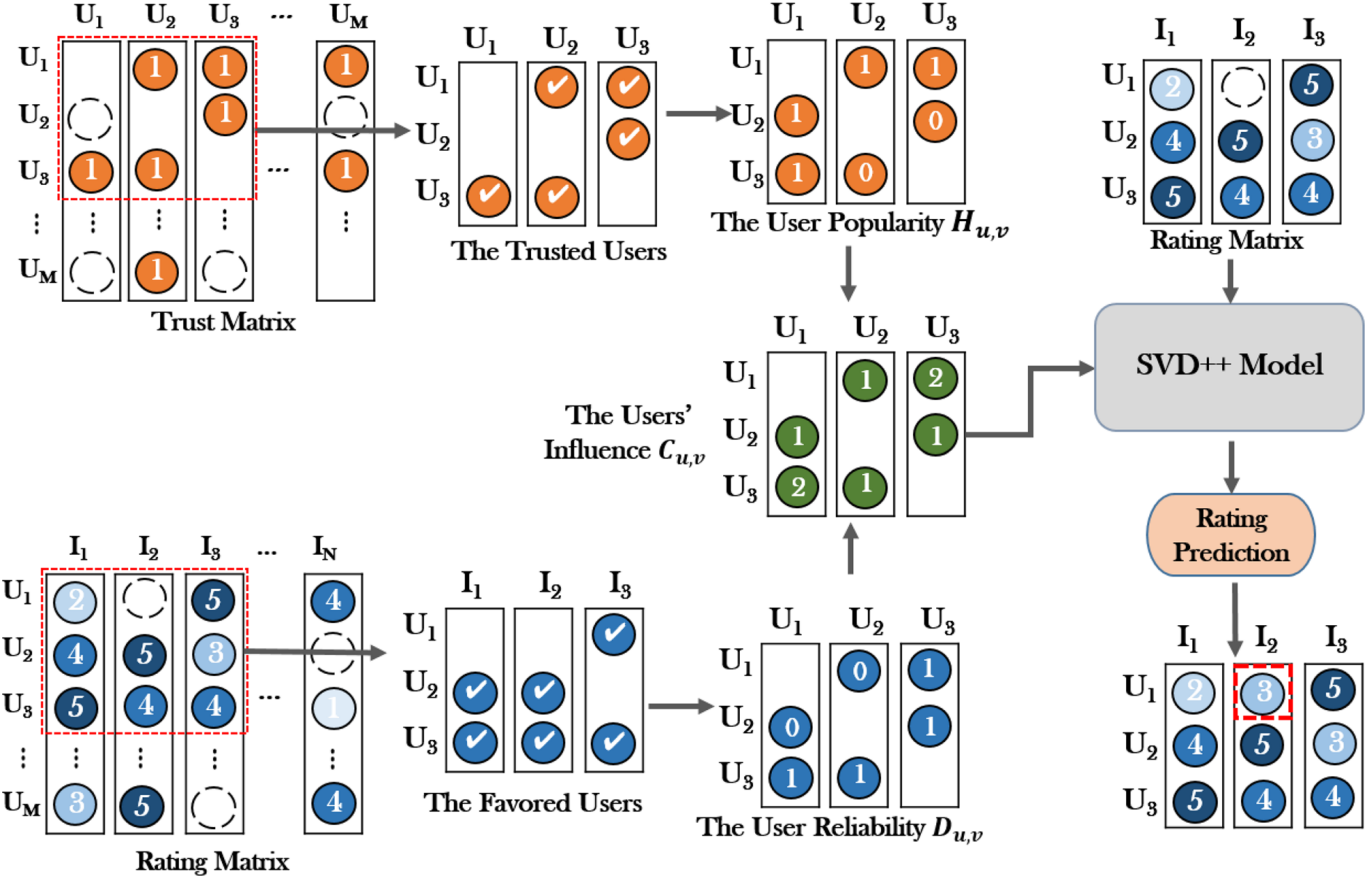
The overview of our proposed rating prediction model (RTRM).

Our proposed model extracts the user reliability and popularity from the user ratings and trust and then integrates them into the recommendation algorithm based on the MF for rating prediction. The overall framework of our proposed rating prediction model is presented in Fig. 1. The user ratings and trust are extracted and analyzed by the Word2vec model^53^, and then the two factors (user reliability and popularity) are integrated into the MF for rating prediction.

## Proposed rating prediction model

First, we introduce the model of analyzing user reliability and popularity from user ratings and trust. Then, we describe our proposed model. Finally, the training process of the model is illustrated.

### User reliability and popularity

In this section, we present a model to analyze user reliability and popularity from user ratings and trust. First, we explain how the User-wise Mutual Information (UMI) between different users is calculated.

Inspired by the application of the word embedding model^54^ in the field of natural language processing, we defined the mutual information among different users as the UMI. Considering a user as a word, all users are a complete word context set. Let D be the set of observed words and context pairs. The ${{U}}{{MI}}\left({a, b}\right)$ between word *a* and context word *b* is calculated as:

1 $${\text{UMI}}\left({a, b}\right)=\log\frac{{{P}}\left({{a}}{, }{{b}}\right)}{{{P}}\left({{a}}\right){*P}\left({{b}}\right)}$$

where ${\text{P}}\left({a,} \, {{b}}\right)$ is the probability of word *a* appearing together with context word *b* in a specific window size (*D*) (${\text{P}}\left({a,} \, {{b}}\right)={\#}\left({a,} \, {{b}}\right)/\left|{{D}}\right|$, $\left|{{D}}\right|$ refers to the total number of words and the words’ context pairs in *D*). Similarly, ${{P}}\left({{a}}\right)$ represents the probability of word *a* appearing in *D* (${{P}}\left({{a}}\right)={\#}\left({{a}}\right)/\left|{{D}}\right|$), and ${P(b)}$ represents the probability of word b appearing in D (${{P}}\left({{b}}\right)={\#}\left({{b}}\right)/\left|{{D}}\right|$). The final value of ${\text{UMI}}\left({a,} \, {{b}}\right)$ can be calculated as follows:

2 $${\text{UMI}}\left({a, b}\right)=\log\frac{{\#}\left({a, b}\right){*}\left|{{D}}\right|}{{\#}\left({{a}}\right){*\#}\left({{b}}\right)}$$

We define a user who rated more than 70% of the highest rating as a strong favorite user of this item^55,56^ (when the highest rating is 5 points, users with a rating of 4 and 5 points are strong users of this item).

**Figure 2:**
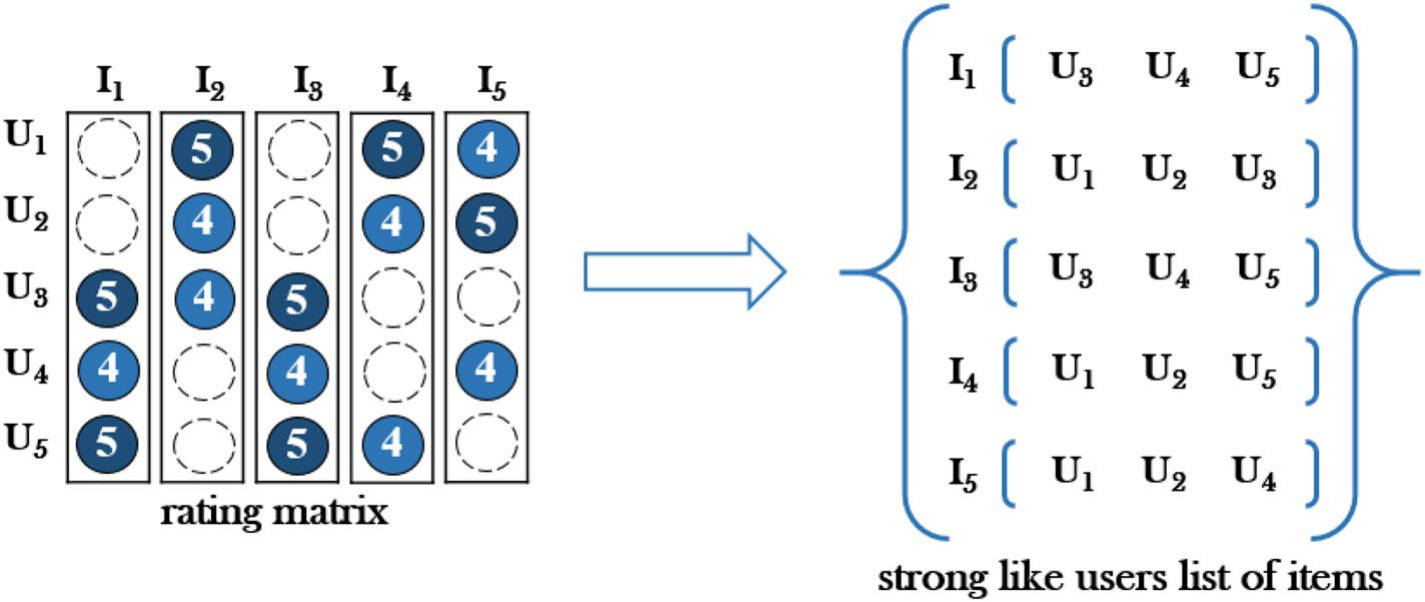
The rating matrix is converted into a list of strong liking users for each item.

Figure 2. shows the process of converting from a rating matrix to a strong liking user list. First, we calculate and extract a strong favorite user collection for each item in the dataset. The total number of times a user is observed and the number of times he or she appears in the list of strong liking users on a given item is expressed as L. The ${\text{UMI}}\left({u,} \, {{v}}\right)$ value between a specific user *u* and another user *v* is calculated as follows:

3 $${\text{UMI}}\left({u,} \, {{v}}\right)= {log} \frac{{\#}\left({u,} \, {{v}}\right){*}\left|{{L}}\right|}{{\#}\left({{u}}\right){*\#}\left({{v}}\right)}$$

By calculating the ${{UMI}}$ of all users and their corresponding users in L, we can form a square matrix of ${{M}} \times {{M}}$, where M is the total number of users in the dataset. The larger the ${{UMI}}\left({u,} \, {{v}}\right)$ value is, the greater the correlation between user *u* and user *v*. In other words, the larger the corresponding filling value in user reliability matrix ${{D}}_{{u,} \, {{v}}}$ is, the higher the reliability of user *u* is for user *v*. The fill value of the matrix ${{{D}}_{{u,} \, {{v}}}}^{{{M}} \times {{M}}}$ is defined as follows:

4 $${{D}}_{{u,} \, {{v}}}={{UMI}}\left({u,} \, {{v}}\right)/{{maxUMI}}\left({u,} \, {{v}}\right)$$

To facilitate the calculation of the model without changing the weights of the values, we use the obtained UMI as the numerator, the maximum value of the UMI as the denominator, and map the impact value between 0 and 1. And the definition of Eq. (6) below is in the same way.

**Figure 3:**
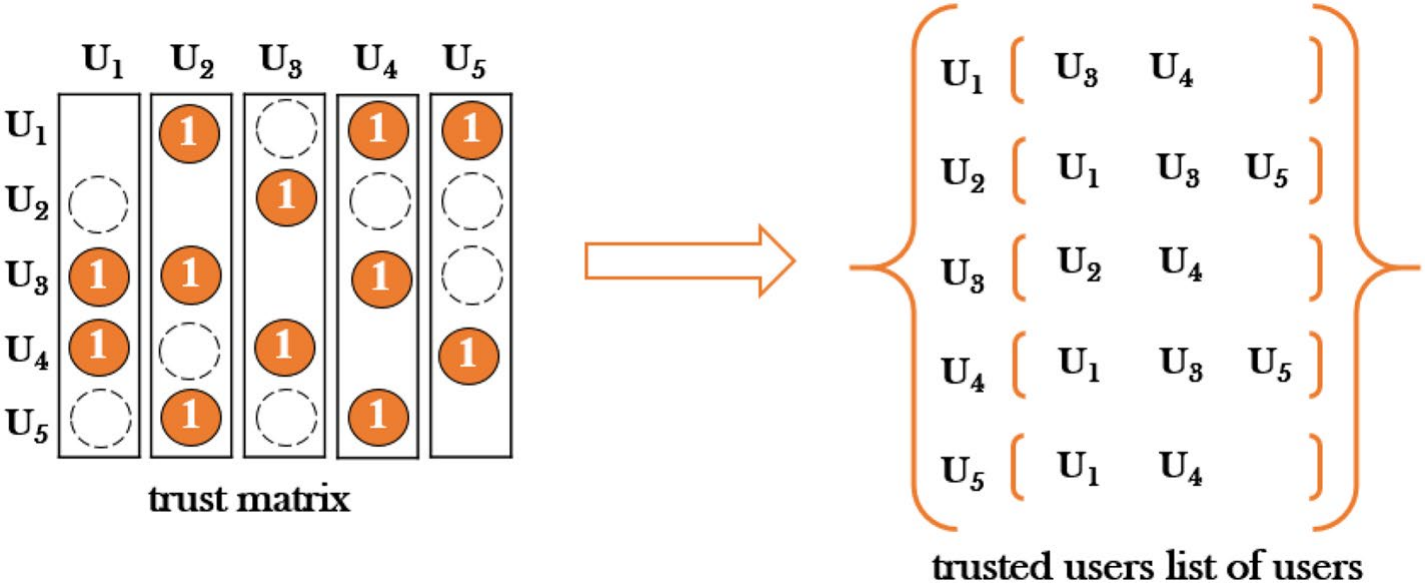
The user trust matrix translates to the user's list of trusted users.

The trust relationship between users is generally represented as the trust matrix ${{O}}_{{M \times M}}$. If there is a trust relationship between users, the corresponding filling value is “1”. If there is no trust relationship, the cell is empty. Similar to the way the user ratings are processed, we transform the user trust matrix into a list of trusted users for each user (Fig. 3.). The more people trust user ${{u}}^{{\prime}}$, the more popular user ${{u}}^{{\prime}}$ is. The relationship between user ${{u}}^{{\prime}}$ and another user ${{v}}^{{\prime}}$ is defined as follows:

5 $${\text{UMI}}\left({{u}}^{{\prime}}{,} \, {{v}}^{{\prime}}\right)=\log\frac{{\#}\left({{u}}^{{\prime}}{,}{ \, {{v}}}^{{\prime}}\right){*}\left|{{L}}^{{\prime}}\right|}{{\#}\left({{u}}^{{\prime}}\right){*\#}\left({{v}}^{{\prime}}\right)}$$

where the total number of times that a particular user appears with his or her trusted users is expressed as ${{L}}^{{\prime}}$. The larger the ${{UMI}}\left({{u}}^{{\prime}}{,}{{v}}^{{\prime}}\right)$ value is, the greater the popularity between user ${{u}}^{{\prime}}$ and user ${{v}}^{{\prime}}$. In other words, the larger the corresponding filling value in the user popularity matrix ${{{H}}_{{{u}}^{{\prime}}{,}{{v}}^{{\prime}}}}^{{{M}} \times {{M}}}$ is, the higher the popularity of user ${{u}}^{{\prime}}$ is for user ${{v}}^{{\prime}}$. The fill value of the matrix ${{{H}}_{{{u}}^{{\prime}}{,}{{v}}^{{\prime}}}}^{{{M}} \times {{M}}}$ is defined as follows:

6 $${{H}}_{{{u}}^{{\prime}}{,} \, {{v}}^{{\prime}}}={{UMI}}\left({{u}}^{{\prime}}{,}{ \, {{v}}}^{{\prime}}\right)/{{maxUMI}}\left({{u}}^{{\prime}}{,} \, {{v}}^{{\prime}}\right)$$

Similarly, ${{maxUMI}}\left({{u}}^{{\prime}}{,}{ \, {{v}}}^{{\prime}}\right)$ represents the maximum mutual information between global users, the calculated mutual information between each two users is compared with the maximum, and the final matrix filling value ranges between {0,1}.

After the above calculation of the user reliability matrix ${{{D}}_{{u,} \, {{v}}}}^{{{M}} \times {{M}}}$ and user popularity matrix ${{{H}}_{{u,} \, {{v}}}}^{{{M}} \times {{M}}}$ between user *u* and user *v* based on the user rating and trust, we obtained the overall influence matrix ${{{C}}_{{u,} \, {{v}}}}^{{{M}} \times {{M}}}$ of user *u* and user *v*, which is defined as follows:

7 $${{{C}}_{{u,} \, {{v}}}}^{{{M}} \times {{M}}}=\max\left\{{\left({{D}}_{{u,} \, {{v}}}{+}{{H}}_{{u,} \, {{v}}}\right)}^{{{M}} \times {{M}}}-\left(\lg\eta\right){1}^{{{M}} \times {{M}}} ,{0}\right\}$$

where $\left({D}_{u, v}+{H}_{u, v}\right)$ represents the sum of the element value corresponding to the position in the matrix ${{{D}}_{{u,} \, {{v}}}}^{{{M}} \times {{M}}}$ and ${{{H}}_{{u,} \, {{v}}}}^{{{M}} \times {{M}}}$. The matrix ${{{D}}_{{u,} \, {{v}}}}^{{{M}} \times {{M}}}$ represents the user-user influence calculated from the ratings data, and the matrix ${{{H}}_{{u,} \, {{v}}}}^{{{M}} \times {{M}}}$ represents the user-user influence calculated from the trust data. And the dimensions and status of the two matrices are the same, so we add the values of the corresponding positions of the two matrices to obtain the total impact matrix ${{{C}}_{{u,} \, {{v}}}}^{{{M}} \times {{M}}}$ between users. The $\eta$ is the parameter used to control the dense degree of the matrix ${{{C}}_{{u,} \, {{v}}}}^{{{M}} \times {{M}}}$*.* Then, $\eta$ and the density degree of the matrix $C_{{u,{\kern 1pt} v}} ^{M  \times M}$ are inversely proportional. In other words, the greater the value of $\eta$, the lower the density degree of the matrix ${{{C}}_{{u,v}}}^{{{M}} \times {{M}}}$ is.

### Rating prediction model

In the recommendation system, the classic formula for rating prediction based on the MF model is as follows:

8 $$ \widehat{R}_{{u,{\kern 1pt} i}}  = \mathop R\limits^{ - }  + B_{u}  + B_{i}  + \left( {P_{u}  + \left| {N_{u} } \right|^{{ - 1/2}} \sum\limits_{{j \in N_{u} }} {Y_{j} } } \right)Q_{i}^{T}  $$

9 $${{e}}_{{u,} \, {{i}}}={{R}}_{{u,} \, {{i}}}-{\widehat{{R}}}_{{u,} \, {{i}}}$$

where ${\widehat{{R}}}_{{u,} \, {{i}}}$ represents user *u*’s rating on item *i* by the algorithm, and $\overline{R}$ represents the mean of the global ratings. ${{B}}_{{u}}$ represents the user bias (the rate habits of a particular user), which is independent of item characteristics. ${{B}}_{{i}}$ represents the item bias (a rating given for a particular item), which is independent of the user interest. For a particular user *u*, the collection of items that it provides the implicit feedback is defined as ${{N}}_{{u}}$. ${{Y}}_{{n}}$ denotes the implicit influence of items rated by user *u* in the past on the ratings of unknown items in the future, and all of the user *u*’s ratings revised values are $\sum\limits_{{{{j}} \in {{N}}_{{{u}}} }} {{{Y}}_{{{j}}} }$. They are usually expressed as ${{Y}}_{{j}}{{{Q}}}_{{i}}^{{T}}$, and ${\left|{{N}}_{{u}}\right|}^{-1/2}$ is introduced to eliminate the difference caused by different ${{N}}_{{u}}$ numbers. ${{e}}_{{u,} \, {{i}}}$ represents the deviation between the real rating and the predicted rating.

By analyzing the overall influence of user *u* on user *v* obtained from user ratings and trust, we defined the objective function of the proposed recommendation model as follows:

10 $$  \begin{aligned}   J\left( {R,P,Q} \right) &  = \frac{1}{2}\sum\limits_{{u,i}} {\left( {e_{{u,i}} } \right)^{2} }  + \frac{\lambda }{2}\left( {P_{{u_{F}^{2} }}  + Q_{{i_{F}^{2} }}  + B_{{u_{F}^{2} }}  + B_{{i_{F}^{2} }}  + \sum\limits_{{j \in N_{u} }} {Y_{{j_{F}^{2} }} } } \right) \\     &  + {\mkern 1mu} \frac{\mu }{2}\sum\limits_{u} {\left\{ {\left( {1^{M}  \times MP_{u}  - \sum\limits_{v} {C_{{u,{\kern 1pt} v}} ^{M} }  \times MP_{v} } \right)\left( {1^{M}  \times MP_{u}  - \sum\limits_{v} {C_{{u,{\kern 1pt} v}} ^{M} }  \times MP_{v} } \right)^{T} } \right\}}  \\  \end{aligned}  $$

where ${{R}}_{{u,} \, {{i}}}$ represents the true rating of user *u* on item *i*. ${{R}}_{{{{u,i}}}}  \in {{R}}_{{{M}}}  \times {{N}}$, where *M* represents the number of users, *N* represents the number of items, and *R* represents the ratings set of the users on the items. We obtained the parameters in Eq. (10) through the gradient descent model^57^; ${{P}}_{{M}} \times {{K}}$ and ${{Q}}_{{K}} \times {{N}}$ represent the potential factor matrix of the users and items, and ${{P}}_{{u}}$ and ${{Q}}_{{i}}$ represent the k-dimensional implicit vector of user *u* and item *i*. The first part of Eq. (10) represents the deviation of the true rating ${{R}}_{{u,} \, {{i}}}$ and the prediction rating ${\widehat{{R}}}_{{u,} \, {{i}}}$, and the second part represents the regular term that is set to prevent the overfitting of the model. The third part shows the influence of the reliability and popularity among users on rating predictions, which means that compared with user *u*, if user *v* has higher reliability and popularity, user *u*'s rating of a certain item *i* will be greatly affected by user *v*.

### Model training

The objective function of our recommendation model is given by Eq. (10). According to the gradient descent model, we obtain the user bias ${{B}}_{{u}}$, the item bias ${{B}}_{{i}}$, the user implicit feedback ${{Y}}_{{j}}$, and the k-dimensional potential factors ${{P}}_{{u}}$ and ${{Q}}_{{i}}$ of users and items. Their gradient is shown as follows:

11 $$ \frac{{\partial J}}{{\partial B_{u} }} = \sum\limits_{i} {e_{{u,{\kern 1pt} i}} }  + \lambda B_{u}  $$

12 $$\frac{{\partial J}}{{\partial}{{B}}_{{i}}}=\sum_{{u}}{{{e}}}_{{u, i}}{+\lambda}{{B}}_{{i}}$$

13 $$ \frac{{\partial J}}{{\partial Q_{i} }} = \sum\limits_{i} {e_{{u,{\kern 1pt} i}} } P_{u}  + \lambda Q_{i}  $$

14 $$ \frac{{\partial {\text{J}}}}{{\partial Y_{u} }} = \sum\limits_{u} {e_{{u,{\kern 1pt} i}} } \left| {N_{u} } \right|^{{ - 1/2}} Q_{i}  + \lambda \sum\limits_{{j \in N_{u} }} {Y_{j} }  $$

15 $$ \frac{{\partial J}}{{\partial P_{u} }} = \sum\limits_{i} {e_{{u,{\kern 1pt} i}} } Q_{i}  + \lambda P_{u}  + \mu \left( {P_{u}  - \sum\limits_{v} {C_{{u,{\kern 1pt} v}} } P_{v} } \right) - \mu \sum\limits_{v} {C_{{u,{\kern 1pt} v}} } \left( {P_{u}  - \sum\limits_{v} {C_{{u,{\kern 1pt} v}} } P_{v} } \right) $$

In addition, the initial values of ${{B}}_{{u}}$ and ${{B}}_{{i}}$ are set as the 0 vector, and the initial values of ${{P}}_{{u}}$ and ${{Q}}_{{i}}$ are obtained from the normal distribution by sampling with the zero mean value. Then, the update process of each parameter is as follows:

16 $${{{B}}_{{u}}}^{\left({{t}}\right)}={{{B}}_{{u}}}^{\left({t} - {1}\right)}{-\gamma}{\partial}{{J}}^{\left({t} - {1}\right)}/{\partial}{{B}}_{{u}}$$

17 $${{{B}}_{{i}}}^{\left({{t}}\right)}={{{B}}_{{i}}}^{\left({t} - {1}\right)}{-\gamma}{\partial}{{J}}^{\left({t} - {1}\right)}/{\partial}{{B}}_{{i}}$$

18 $${{{Y}}_{{j}}}^{\left({{t}}\right)}={{{Y}}_{{j}}}^{\left({t} - {1}\right)}{-\gamma}{\partial}{{J}}^{\left({t} - {1}\right)}/{\partial}{{Y}}_{{j}}$$

19 $${{{P}}_{{u}}}^{\left({{t}}\right)}={{{P}}_{{u}}}^{\left({t} - {1}\right)}{-\gamma}{\partial}{{J}}^{\left({t} - {1}\right)}/{\partial}{{P}}_{{u}}$$

20 $${{{Q}}_{{i}}}^{\left({{t}}\right)}={{{Q}}_{{i}}}^{\left({t} - {1}\right)}{-\gamma}{\partial}{{J}}^{\left({t} - {1}\right)}/{\partial}{{Q}}_{{i}}$$

In this paper, the learning rate *γ* of the model is set as 0.005. To ensure the objective function keeps decreasing, the total number of iterations is set as 10,000, and the pre-stop identifier is set (jumping out of the iteration when the gradient is no longer falling).

## Experiments

In this section, several experiments are carried out on the datasets of FilmTrust and Epinions to contrast the rating predicted performance of the RTRM to other state-of-the-art models. In the following chapters, we present detailed information on the experimental datasets, evaluation metrics, compared algorithms, and experimental results.

### Experimental datasets

Since this paper focuses on social recommendation, two open datasets (FilmTrust and Epinions) containing user ratings and trust were used to test the performance of the recommendation algorithms, including the proposed model in this paper, in rating predictions and cold starts. Specifically, the FilmTrust dataset was collected by Guo et al. from the FilmTrust website^58^, which is a film recommendation website based on the trust relationship. Users can rate films according to their preferences and build a one-way trust relationship. The Epinions dataset was collected by Massa et al. from the Epinions website^54^, which provides comparison information of various goods for users to rate, and users can also add trusted users to build directed social networks. The rating ranges of the different datasets are different. FilmTrust's rating range is [0.5, 4], and the step size is 0.5. The Epinions' rating range is [1,5], and the step size is 1. Detailed statistics for the above two datasets are shown in Table 3, where RDensity represents the dense degree of ratings data, $\overline{m}$ represents the average ratings of the individual users, $\overline{n}$ represents the mean rated users of a single item, SDensity represents the dense degree of the users’ trust, and $\overline{s}$ represents the average number of the users’ trust.

**Table 3:** Statistics of Experimental Datasets.

	Users	Items	Ratings	Rdensity (%)	Trust	SDensity (%)
FilmTrust	1508	2071	35,497	1.14	1853	0.069
Epinions	40,163	139,738	664,824	0.011	487,183	0.021

### Evaluation metrics

To measure the accuracy of rating predictions, this paper adopts two commonly used evaluation indicators in recommendations: Root Mean Squared Error (*RMSE*) and Mean Absolute Error (*MAE*). These two indexes measure the accuracy of the recommendation results by calculating the error between the real rating and the predicted rating. The smaller the value, the higher the recommendation accuracy. They are defined by the following formulas:

21 $${{RM}}{SE} = \sqrt{\frac{1}{{{T}}}\sum_{{u,} \, {{i}}}{\left({{R}}_{{u,} \, {{i}}}-{\widehat{{R}}}_{{u,} \, {{i}}}\right)}^{2}}$$

22 $${MAE} = { }\frac{1}{{{T}}}\sum_{{u,} \, {{i}}}\left|{{R}}_{{u,} \, {{i}}}-{\widehat{{R}}}_{{u,} \, {{i}}}\right|$$

Among them, *T* represents the number of ratings in the test set, ${{R}}_{{u,} \, {{i}}}$ represents the true rating value, and ${\widehat{{R}}}_{{u,} \, {{i}}}$ represents the prediction rating value.

### Compared algorithms

In this paper, a series of experiments were carried out to test our proposed model for evaluating the performance of the comprehensive and cold start users. Among the many recommendation models based on social relationships, we selected several relatively new and representative models. Table 4. describes the similarities and differences between the selected comparison models. A brief description of these models is as follows:

**Table 4:** Similarity and difference between the selected comparison models.

Models	Similarity	Difference
SoRec	a. Matrix Factorization model b. Trust-based model c. Rating Prediction model d. Factorization of Rating Matrix and Trust Matrix Simultaneously	The user trust matrix was add to the original probability matrix factorization recommendation model
SocialMF		The weighted average of the feature vectors of users' trusted friends was added to the matrix factorization recommendation model
SoReg		The result of regularizing user feature vector using the trust was added to the matrix factorization recommendation model
LOCABAL		The local and global social information was added to the matrix factorization recommendation model
TrustSVD		The user's explicit trust which was treated as implicit feedback as the rating was added to the matrix factorization recommendation model
MFC		The regularized social information of overlapping communities was added to the matrix factorization recommendation model
SoDimRec		The user social dimension information was added to the matrix factorization recommendation model
CUNE		The implicit and reliable social information extracted from user feedback was added to the matrix factorization recommendation model

**SoRec**^55^ The user social relation matrix was added to the recommendation model of the original PMF, and it was decomposed into a user characteristic matrix and a social characteristic matrix to improve the accuracy of rating prediction.

**SocialMF**: A new social recommendation model is based on the trust transmission mechanism in social networks.

**SoReg**^56^ This social regularization recommendation model is the first to propose the difference between trust relationships and friend relationships. This model believes that the establishment of trust relationships depends on users having similar interests and preferences, while the establishment of friend relationships depends more on users' social relationships in the real world.

**LOCABAL**^57^ A social recommendation model that combines the rating matrix and the social relation matrix using local and global social relations.

**TrustSVD** In this model, the explicit trust relationship between users and the rating information are regarded as implicit feedback information, and social feedback information is added to the original model to reconstruct the rating prediction model.

**MFC** A recommendation model of regularization and integration of social information in overlapping communities, which considers that users classified into the same communities have similar tastes.

**SoDimRec** A recommendation model based on the social dimension is proposed based on the assumption that social relations have isomorphism in the existing recommendation models.

**CUNE**^58^ A recommendation model that extracts the implicit and reliable social information from user feedback and identifies the first *k* semantic friends for each user to improve the accuracy of rating predictions.

### Performance comparison

In this paper, the model is trained and tested by the fivefold cross validation method. Specifically, the dataset is divided equally at random, with 80% of the dataset used as the training set and 20% as the test set in each test. Five experiments can ensure that all data are tested, and the final test result is the average of the results of 5 experiments.

We compare our proposed recommendation model with the above comparison algorithms. We set the dimension *K* of the potential factors of ${{P}}_{{u}}$ and ${{Q}}_{{i}}$ to 10 in our objective function and set the number of rated users less than or equal to 5 to the "cold start users". ${\lambda}$ is the parameter that prevents the model from overfitting, and ${\mu}$ is the parameter that optimizes the degree of impact to users. Specifically, we first fix ${\lambda}=1$, tuning ${\mu}$ in [10^−2^, 10^−1^,1,10]. Then, with the best ${\mu}$, we tune ${\lambda}$ in [10^−2^, 10^−1^,1,10]. Note that if the optimal value was found in the boundary, we further extend the boundary to explore the optimal setting. After many iterations of testing, the parameters ${\lambda}=0.65$ and ${\mu}=0.4$ are assigned to the FilmTrust dataset and the parameters ${\lambda}=0.9$ and ${\mu}=0.5$ are assigned to the Epinions dataset. Table 3. shows our model compared with the above model in MAE and RMSE for all users and for cold start users.

In Table 5, we show the performance of all the comparison models and our proposed model on the two datasets. The first part is the comprehensive performance of users, and the second part is the cold start performance. We used a bold font to represent the best performance on each of the two datasets, and the symbol * is used to indicate the second-best performance. The TrustSVD algorithm has achieved good results (the FilmTrust dataset on cold start performance is an exception) regardless of *RMSE* or *MAE*. The main reason is that the TrustSVD model adds implicit social feedback information to re-express the user feature matrix and decomposes the social relationship matrix to form the user feature matrix and the social feature matrix. It is clear that our approach (RTRM) is superior to the other models on each metric for both datasets. Relative to the second-best performance, we show the improvement of our model: (1) The *RMSE* index of the RTRM improved by 2.441–2.661%, and the *MAE* index improved by 1.689–2.740% in terms of the overall performance. (2) The *RMSE* index of the RTRM is decreased by 3.132–4.748%, and the *MAE* index is decreased by 3.905–4.050% in terms of cold start user performance. The above experimental results show that our model has a high accuracy of rating predictions and can effectively alleviate the cold start problem.

**Table 5:** Performance comparisons in all user and cold start user test views, where bold indicates the best performance of all other methods, and the "improved" column indicates the relative improvement of our RTRM method relative to the best results.

All	FilmTrust		Epinions		Cold Start	FilmTrust		Epinions	
Metrics	RMSE	MAE	RMSE	MAE	Metrics Models	RMSE	MAE	RMSE	MAE
Models									
SoRec	0.8369	0.6486	1.2451	0.9368	SoRec	0.8911	0.7204	1.2906	1.0762
SocialMF	0.8316	0.6481	1.2903	0.9637	SocialMF	0.8704	0.6821	1.3116	1.0541
SoReg	0.8444	0.6431	1.2802	0.9609	SoReg	0.9080	0.7225	1.2914	1.0621
LOCABAL	0.8297	0.6519	1.1316	0.8477	LOCABAL	0.8402*	0.6469*	1.2074	0.8973
TrustSVD	0.8192*	0.6349*	1.0901*	0.8344*	Trust-SVD	0.8476	0.6514	1.1875*	0.8628*
MFC	0.8198	0.6496	1.1263	0.8360	MFC	0.8649	0.6713	1.2101	0.8798
SoDimRec	0.8193	0.6417	1.1292	0.8408	SoDimRec	0.8721	0.6775	1.2029	0.8785
CUNE	0.8390	0.6608	1.1391	0.8604	CUNE	0.8451	0.6528	1.2186	0.8810
**RTRM**	**0.7992**	**0.6175**	**1.0609**	**0.8203**	**RTRM**	**0.8003**	**0.6207**	**1.1503**	**0.8291**
**Improved**	**2.441%**	**2.740%**	**2.678%**	**1.689%**	**Improve**	**4.748%**	**4.050%**	**3.132%**	**3.905%**

## Discussion

In addition to the comprehensive performance and the cold start user performance comparison given in Table 5, we also discuss four aspects that affect the model performance: *A.* the influence of the potential vector dimension *K* of users and items on model performance; *B.* the influence of the factors between users on model performance; *C.* the influence of the density of factor matrix ${C}_{u,v}$ on model performance; and *D.* the influence of the training data volume on model performance. In this section, to further illustrate our proposed algorithm’s performance, we use the *A-RMSE* and *A-MAE* to represent the comprehensive performance and the *C-RMSE* and *C-MAE* to represent the cold start user performance.

### The influence of the potential vector dimension K of users and items on model performance

Because the TrustSVD model fully takes the explicit and implicit effects of the rating and trust into account in the recommendation process, it is relatively stable when compared with the changes in the dimension of the potential feature vectors of users and items, and it performs well in the rating predictions on all users and the cold start users. Table 6. shows the comparison between our proposed model and the TrustSVD model in the performance of rating predictions on all users and cold start users in the context of changes in the dimension of potential feature vectors for the users and items. The experimental results show that compared with the TrustSVD model, our proposed model has a larger overall improvement, and the results do not fluctuate greatly depending on the dimension.

**Table 6:** Performance comparison between Trust-SVD and our methods in the case of changes in user and item potential feature dimensions K.

Trust-SVD vs RTRM (All)	FilmTrust		Epinions		Trust-SVD vs RTRM (Cold Start)	FilmTrust		Epinions	
	K = 5	K = 10	K = 5	K = 10		K = 5	K = 10	K = 5	K = 10
A-RMSE	0.8213	0.8192	1.0981	1.0901	C-RMSE	0.8512	0.8412	1.1967	1.1875
	**0.8102**	**0.7992**	**1.0663**	**1.0609**		**0.8023**	**0.8003**	**1.1607**	**1.1503**
A-MAE	0.6261	0.6349	0.8394	0.8344	C-MAE	0.6516	0.6469	0.8736	0.8628
	**0.6195**	**0.6175**	**0.8287**	**0.8203**		**0.6226**	**0.6207**	**0.8341**	**0.8291**

### The influence of the factors between users on model performance

**Figure 4:**
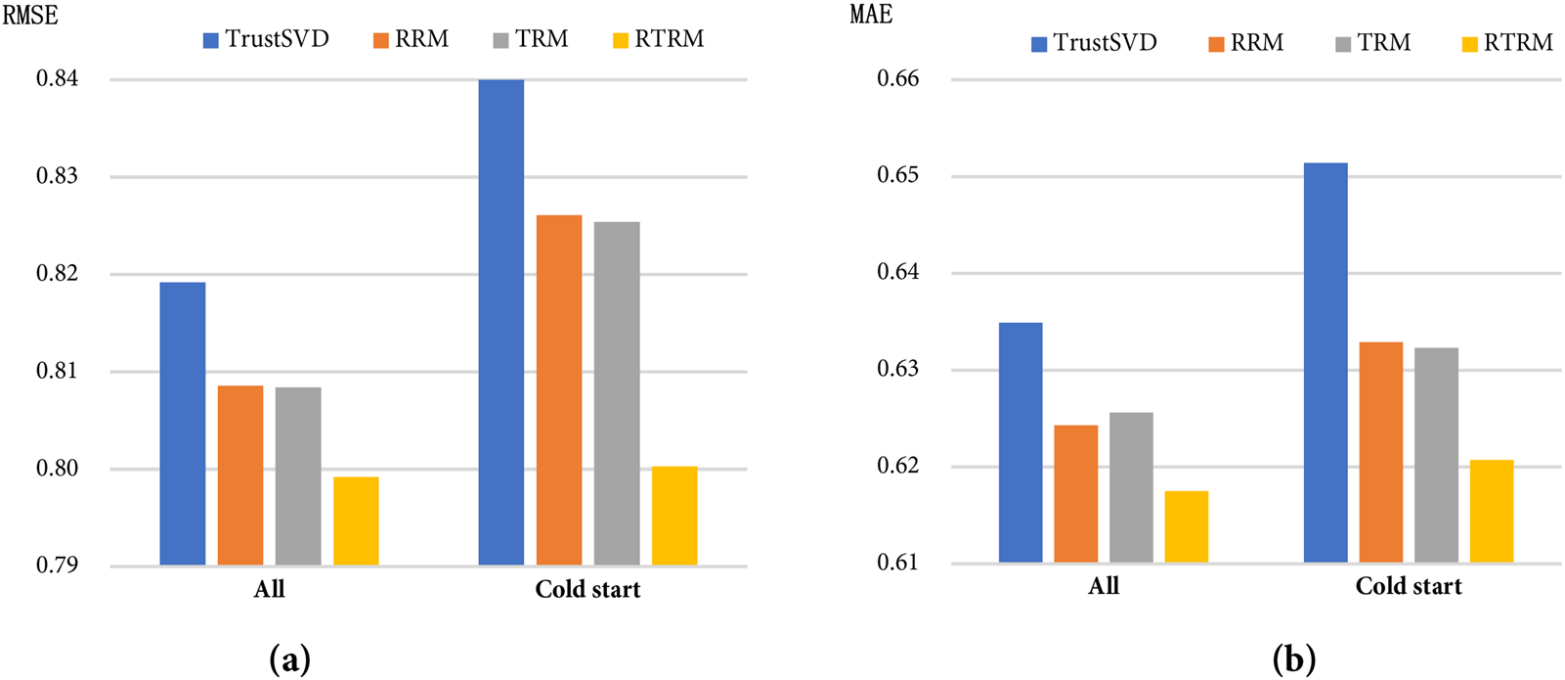
The impact of each factor between users on the experimental results in the FilmTrust dataset.

To verify the validity of the influence factors between the two users proposed by our model, we conducted a series of experiments, and the results are shown in Fig. 4. Given the TrustSVD model's good performance in the rating predictions of all users and cold start users, we use the TrustSVD model as a comparative experiment in this section. *RRM* represents a rating prediction model that only considers the reliability among users. *TRM* represents the rating predictions model that only considers the popularity among users. *RTRM* represents the rating prediction model that takes inter-user reliability and inter-user popularity into account at the same time. From Fig. 4, we can find that either of the above two factors is effective, but combining the two factors is the best way to improve the accuracy of rating predictions and alleviate the cold start problem.

### The influence of the density of factor matrix ${{\varvec{C}}}_{{\varvec{u}},{\varvec{v}}}$ on model performance

**Figure 5:**
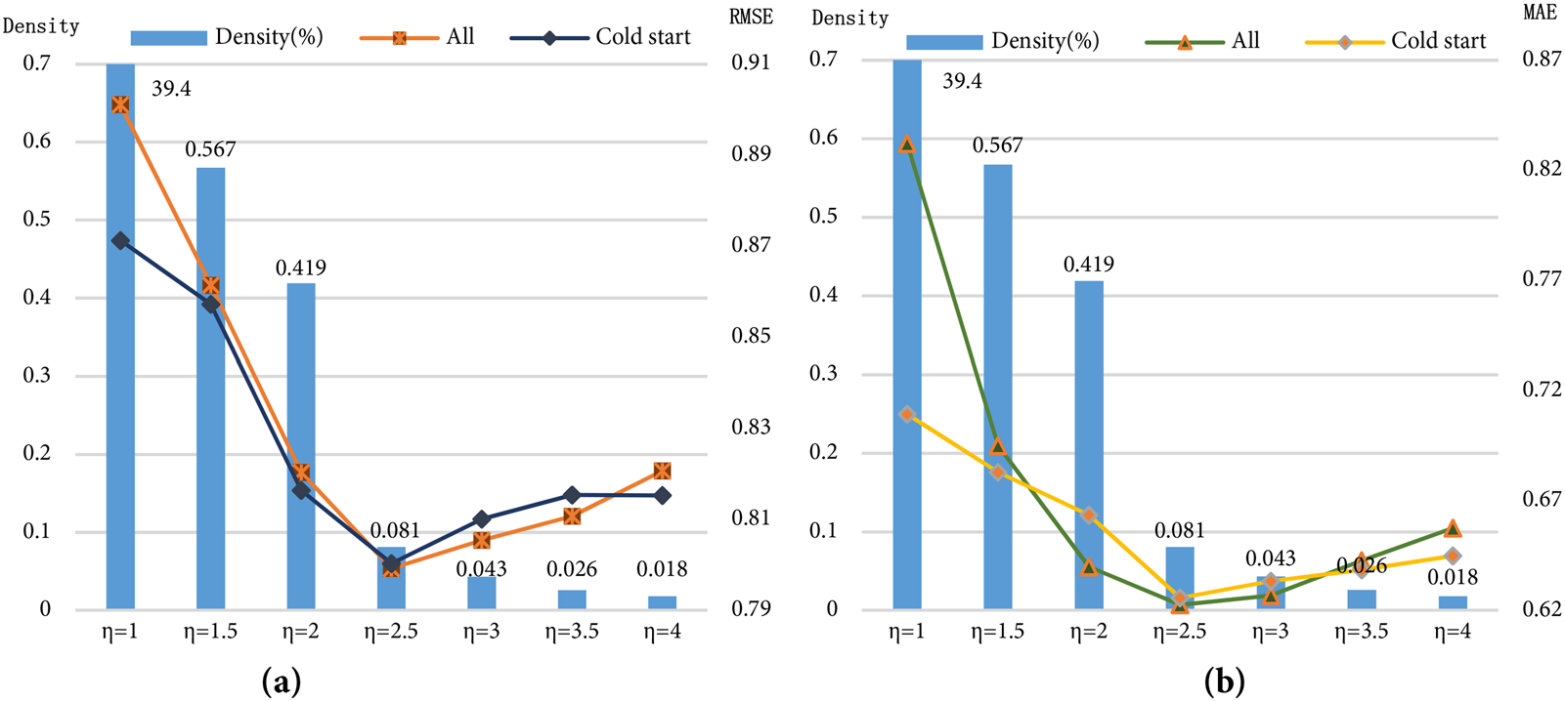
The impact of the density of ${C}_{u,v}$ on the experimental results in the FilmTrust dataset.

The influence among users defined in this paper is determined by the user reliability and popularity obtained above, as shown in Eq. (7). To measure the influence of the density of the factor matrix among users on the model performance, we change the parameters of the threshold *η*'s value of Eq. (7) to control the matrix ${{C}}_{{u,v}}{^{\prime}}s$ density, and the parameter *η* is larger when the density degree of the matrix ${{C}}_{{u,v}}$ is lower; the model starts to perform better. However, as the parameters *η* increase and the density degree of the matrix ${{C}}_{{u,v}}$ decreases, the performance of the model is not always improved. As shown in Fig. 5, when parameter *η's* value is 2.5 and the density degree of the matrix ${{C}}_{{u,v}}$ is 0.081%, the performance of the model is optimized.

### The influence of the training data volume on model performance

We used the FilmTrust dataset to study the influence of the amount of training data on the experimental results, and the results are shown in Table 7. We reduce the amount of training data during model training randomly. For example, 60% of the training data means that we randomly select 60% from the total ratings data for the training of the model. The results show that the change in the training data has little influence on the experimental results, but with the decrease in the training data, the comprehensive and cold start performance of the model also decrease.

**Table 7:** The impact of less training data on performance in the FilmTrust dataset.

	30% Training	40% Training	50% Training	60% Training	70% Training	80% Training
A-RMSE	0.8295	0.8231	0.8196	0.8109	0.8015	**0.7992**
A-MAE	0.6357	0.6309	0.6283	0.6208	0.6194	**0.6175**
C-RMSE	0.8289	0.8215	0.8179	0.8102	0.8051	**0.8003**
C-MAE	0.6428	0.6386	0.6325	0.6291	0.6254	**0.6207**

## Conclusion

In this paper, we propose a recommendation model based on user ratings and trust. Then, the user internal factors are integrated into a MF model to improve the accuracy of rating predictions and alleviate the cold start problem. Because the current social recommendation model only relies on user trust and ignores the influence of user preference information contained in the user ratings, the social relations among users mined by the model lack sufficient effectiveness. We calculated the user-wise mutual information among different users based on the user ratings and trust to obtain the user reliability and popularity represented by the form of the matrix and integrated them into the MF algorithm as a factor affecting the relationship between users. We performed a series of experimental research on two datasets. The experimental results show that the overall performance of our model is at least 2.441% lower in the RMSE index and 1.689% lower in the MAE index. In terms of cold start user performance, the RMSE index decreased by at least 3.132%, and the MAE index decreased by at least 3.905%. This indicates that our proposed model is effective in improving the accuracy of rating predictions and alleviating the cold start problem.

In future work, we will explore more user social behaviors, such as user comment information, the potential preference of user rating behavior and other factors, to further improve the accuracy of rating predictions and the quality of recommendations.
